# A Theoretical Framework for How We Learn Aesthetic Values

**DOI:** 10.3389/fnhum.2020.00345

**Published:** 2020-09-11

**Authors:** Hassan Aleem, Ivan Correa-Herran, Norberto M. Grzywacz

**Affiliations:** ^1^Interdisciplinary Program in Neuroscience, Georgetown University, Washington, DC, United States; ^2^Department of Neuroscience, Georgetown University, Washington, DC, United States; ^3^Facultad de Artes, Universidad Nacional de Colombia, Bogotá, Colombia; ^4^Department of Psychology, Loyola University Chicago, Chicago, IL, United States; ^5^Department of Molecular Pharmacology and Neuroscience, Loyola University Chicago, Chicago, IL, United States

**Keywords:** aesthetics (as scholarly discipline), reinforcement leaning, art, motivation, preference, computational modeling

## Abstract

How do we come to like the things that we do? Each one of us starts from a relatively similar state at birth, yet we end up with vastly different sets of aesthetic preferences. These preferences go on to define us both as individuals and as members of our cultures. Therefore, it is important to understand how aesthetic preferences form over our lifetimes. This poses a challenging problem: to understand this process, one must account for the many factors at play in the formation of aesthetic values and how these factors influence each other over time. A general framework based on basic neuroscientific principles that can also account for this process is needed. Here, we present such a framework and illustrate it through a model that accounts for the trajectories of aesthetic values over time. Our framework is inspired by meta-analytic data of neuroimaging studies of aesthetic appraisal. This framework incorporates effects of sensory inputs, rewards, and motivational states. Crucially, each one of these effects is probabilistic. We model their interactions under a reinforcement-learning circuitry. Simulations of this model and mathematical analysis of the framework lead to three main findings. First, different people may develop distinct weighing of aesthetic variables because of individual variability in motivation. Second, individuals from different cultures and environments may develop different aesthetic values because of unique sensory inputs and social rewards. Third, because learning is stochastic, stemming from probabilistic sensory inputs, motivations, and rewards, aesthetic values vary in time. These three theoretical findings account for different lines of empirical research. Through our study, we hope to provide a general and unifying framework for understanding the various aspects involved in the formation of aesthetic values over time.

## Introduction

Our aesthetic preferences are an important part of our lives because they shape our decision making and consequently our personality (Skov, 2010). We define ourselves both as individuals and as parts of larger groups through our likes and dislikes (Brown and Dissanayake, 2009). How exactly do these individual preferences come about? Currently, little is known about how preferences form early on and what happens to them throughout our lives. Understanding this process of preference formation has important implications not just for aesthetics, but for philosophy, psychology, neuroscience, marketing, and many other fields. Indeed, philosophy was likely the first discipline to ponder this question (Sartwell, 2012). Philosophers have long wondered whether beauty is shared (universal) or in the eye of the beholder (individual)? We discuss this philosophical question in greater detail elsewhere but touch on it briefly here to frame our work. In our earlier publication, we argue that one can think of universal aspects of preference as innate, formed due to evolutionary pressures (Aleem et al., 2019). Examples include preferences for round contours, symmetry, and contrast. Such preferences are likely present at birth across all populations (Göksun et al., 2014). Here, we are instead more interested in the learned aspects of aesthetic preferences. Specifically, how do individualized aesthetic preferences form under constraints from our environment and experience? How do existing universal aspects of these preferences undergo individual and context-specific changes?

Aesthetic preferences form early on in life, such that by preschool age, children already show idiosyncrasies of their cultures (Senzaki et al., 2014). Furthermore, these preferences continue to evolve over our lifetimes (Park and Huang, 2010). What mechanisms underlie this lifetime evolution? Many of the existing frameworks of aesthetics do not explicitly consider the time-course of preferences. However, several frameworks stress its importance implicitly by focusing on time sensitive variables such as learning and exposure. For example, Leder et al. stress the importance of familiarity, which has been shown to influence preference over time (Leder et al., 2004). However, the framework proposed by Leder et al. is primarily concerned with understanding the aesthetic experience as it plays out, not how preferences form. A closer perspective comes from Vessel and colleagues, who develop an associative theory of aesthetics (Biederman and Vessel, 2006; Vessel and Rubin, 2010). In their view, aesthetic preferences are shaped by associative experiences over our lifetimes. However, their theory focuses primarily on reasons for shared versus individual tastes, not the dynamics of preferences over time *per se*. Several other theories and empirical findings have implications for temporal aspects of aesthetics (see section “Discussion”), but a framework specifically dedicated to understanding this aspect is so far missing.

What should a framework that aims to understand the dynamics of preferences over-time look like? We have constrained our search for such a framework to be within the general principles of neuroscience. Moreover, we have avoided frameworks in which aesthetic values are formed by specialized mechanisms, but rather have focused on known and existing circuitry. A relevant meta-analysis of neuroimaging studies supports this viewpoint for our framework (Brown et al., 2011). These authors analyzed commonalities of aesthetic appraisal across multiple sensory modalities. The results show generalized mechanisms for appraisal centered around a reward-based learning circuit. The central importance of reward is further bolstered by many other imaging studies of aesthetics and appraisal (Lacey et al., 2011; Vartanian and Skov, 2014; Wang et al., 2015). These studies suggest that a reward-based learning mechanism, likely, reinforcement learning, is fundamental to any framework for understanding how aesthetic preferences form. However, these studies and the results from the meta-analysis by Brown et al. (2011), suggest that many factors influence this process of reward-based learning. For example, these factors include interoceptive inputs such as motivations and exteroceptive inputs such as the statistics of sensory stimuli (Brown et al., 2011).

How can a reasonable mechanism of reinforcement learning account for aesthetic individuality? Our individual motivations can greatly influence how we interact with the environment and what decisions we make, thereby having a direct effect on our preferences (Nelson and Morrison, 2005). Motivations can influence reward, for example, activation in reward related regions in the brain in response to certain foods is greatly modulated by food specific satiation (Howard and Kahnt, 2017). Similarly, Brown et al., consider internal drives, or motivations as a key factor in aesthetic appraisal. Since such motivations are individual, they can help account for individuality (Silvia et al., 2009). Therefore, this suggests that motivation may modulate learning of aesthetic preferences. Next, we consider an important factor in aesthetic learning not explicitly addressed by Brown et al., that is, the statistical nature of inputs. Evidence suggests that our perception of incoming inputs is statistical in nature (Pouget et al., 2013). Sensory inputs show many statistical properties that convey useful information which can influence aesthetic preferences. For example, preference for facial symmetry has been shown to be modulated by the presence of pathogen cues (Little et al., 2011). This statistical nature also accounts for differences in preferences amongst cultures, as they impose contingencies on rewards through value systems (Park and Huang, 2010). Finally, internal states such as motivation are also statistical, because we act according to states that vary across time (for example, hunger, tiredness, and sex drive) (Craig, 2009). Sensory inputs, rewards, and motivation do not form a comprehensive list, since a theoretical framework for the learning of aesthetic values is not complete without accounting for semantics, expertise, and much more. However, our framework may capture some of the essential components of aesthetic learning and thus, helps us focus on a simpler model that raises testable predictions. By keeping the model general, we leave ample room for further modifications and increase in complexity.

Following the guidelines listed above, we developed a theoretical framework and a related computational model to investigate the formation and dynamics of aesthetic preferences. The model focuses on visual aesthetic preferences, but our interests go beyond vision or art *per se*. Instead, we are interested in a theoretical framework that is general to the many different domains of preferences. A detailed description of our framework is presented in section “Theoretical Framework.” In turn, section “Materials and Methods” develops the model and described methods for computer simulations of this model. We used these simulations and mathematical analyses to test the following questions: First, we investigated whether aesthetic values would show a dynamic time course, possibly with multiple stages. Moreover, we considered whether these values would be stochastic due to the probabilistic nature of the inputs. Second, we explored how the contingent probabilities of the different variables could lead to a segregation of different trajectories, possibly mimicking different cultures. Third, we investigated whether the individual specific motivation variable would lead to further partitioning of learning trajectories, leading to individuality.

## Theoretical Framework

We have split the description of the theoretical framework into two subsections, general and mathematical. The general section has a description of the ideas without any equations. Our goal here is to help the reader understand the elements of the theoretical framework at an intuitive level. In turn, the mathematical section lays out the equations used to specify the framework precisely. The general description section may allow some readers to skip the equations and go directly to the Results.

### General Description of the Theoretical Framework

**FIGURE 1:**
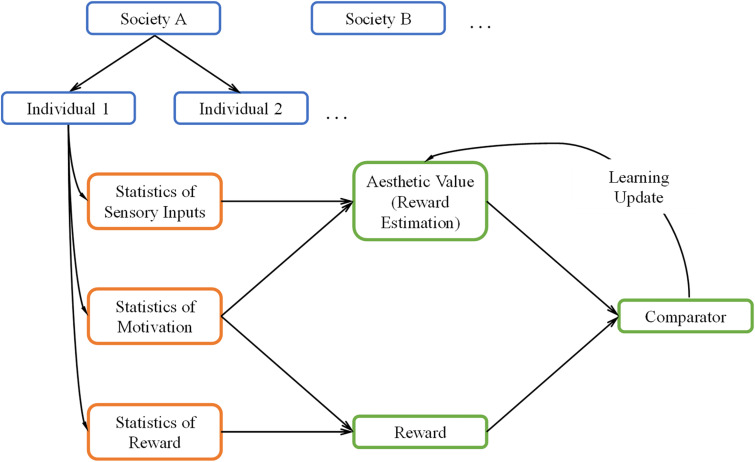
A Schematic overview of our theoretical framework. The framework uses the core reinforcement-learning circuitry (green boxes) with three kinds of inputs (orange boxes). These inputs are statistical, and are both external (sensory inputs and rewards) and internal (motivation) to the brain. The statistics are conditional on each individual and the society of origin of the individual (blue boxes). We postulate the aesthetic value is equivalent to the statistically estimated reward in the reinforcement-learning process.

A general overview of our theoretical framework can be seen in Figure 1. The green boxes in this figure illustrate the core reinforcement-learning system. We discuss it briefly here but see Sutton and Barto (2018) for an extensive overview. In typical reinforcement learning, the system first receives inputs from the external world and from the body (orange boxes). The system then uses these inputs to form an internal model to estimate the reward when taking some action (“Reward Estimation” box), commonly referred to as value. When rewards arrive (“Reward” box), they are compared with the estimated reward (“Comparator” box). If there is a mismatch, the system “learns” by updating the parameters of the internal model. This update allows the system to achieve its goal of producing better reward predictions in the future.

While we based our aesthetic-learning theoretical framework largely on reinforcement learning, our framework has four notable extensions, which make it noteworthy:

First, we propose that the estimate of reward is equivalent to aesthetic value. To help understand this proposal, consider the following example. Imagine a person looking at an apple and smelling it, trying to decide whether to eat it. From the information that the sensory systems collect from the apple, the person makes a prediction about the rewards gained by eating the apple, for example, how sweet and nutritive it is. Then, if the person eats it, their brain will compare actual rewards and its predictions, in updating its model of apples if necessary. Hence, their brain learns that certain statistical properties of apples, for example, shape or color can inform the prediction on their value, which guides the preference for them. Now imagine that the same individual gazes at a painting of an apple. Since some of the same statistical signals may be present in the painting, a similar prediction of reward, or value, is still generated. We propose that the previously learned value will still influence the experience of viewing the painting. If the statistics were previously rewarding, the painting will also elicit high value and generally elicit preference to a similar degree. Therefore, the previous learned value is converted into an aesthetic value.

Second, we incorporate the concept of motivation within the reinforcement-learning circuitry. Motivation, by our definition, is somewhat akin to *policy* (Averbeck and Costa, 2017). It refers to the internal drive of an individual, representing their likelihood to act given an input. For example, if an individual is not hungry, this person will not have the motivation to try a certain food and therefore not learn about its value. However, motivation is not limited to acts of consumption. Experimental interventions can increase the “need” for abstract concepts such as complexity or cognitive closure (Tinio and Leder, 2009; Steciuch et al., 2019). Furthermore, one can choose whether to engage mentally with a work of art if they are motivated. In our framework, motivation is probabilistic, varying from one moment to another, a behavior generally reflecting findings of interoceptive states in humans (Craig, 2009). How does motivation ultimately affect learning? In the simplest manner, motivation controls the rate of learning by slowing it down or accelerating it when the motivation is low or high, respectively. More generally, motivation may affect the learning of certain aesthetic values. For example, an individual who rarely eats fruits may not learn a high aesthetic value for inputs related to fruit.

Third, both interoceptive and exteroceptive inputs to the theoretical framework are statistical (orange/blue boxes in Figure 1). The statistical distributions of these inputs should have significant effects on the learning of values. In detail, statistical interoceptive inputs reflect the variation of motivations across individuals and over time in a single individual (blue boxes). On the other hand, the exteroceptive inputs correspond to both rewards and sensory signals (orange boxes). The statistical nature of these signals reflects the variations of the external world and how different individuals experience it (blue boxes). Continuing our example above, an apple’s sensory signals would be about smell, shape, color, and taste, the reward would be about calories and vitamins, while the interoceptive signals would be the appetite to eat it. The statistical nature of our theoretical framework has three important implications: One, it allows us to generalize over variations of individuals and objects. Two, it ensures that the interoceptive and exteroceptive signals vary stochastically over time, which better approximates real-world conditions. Three, in real life, the statistics of rewards and sensory signals are often correlated, for example, the color and shape of an apple is an indicator of its ripeness. In turn, these attributes influence internal states, for example, a ripe looking apple will be more appetizing. We can model these relationships by using probabilistic distributions for these variables.

Fourth, the inputs to our theoretical framework (orange boxes) depend not only on individuals but also across societies (blue boxes). Therefore, we propose the existence of a parameter space whose values are different across groups (nations, cultures, societies). This means that the distributions of individual statistics are largely conditional on these external parameters. These parameters may specify ecological differences, for example, differences in climates, genetic predispositions, or exposure to diseases (Little et al., 2007; Sorokowski et al., 2014). The social parameters may also specify cultures values, for example, different rewards for certain colors or styles (Masuda et al., 2008; Park and Huang, 2010). By setting the model in the context of social and environmental backgrounds, we can approximate how different societies and cultures form distinct aesthetic values.

In sum, our model begins from a basic circuitry of reward-based learning. Inspired by empirical findings, we expand on this circuitry to include probabilistic inputs, internal drives, and other external contingencies. The combination of these factors allows our model to account for a range of phenomena from the societal all the way to the individual level.

### Mathematical Description of the Theoretical Framework

Let the sensory inputs be N dimensional, with the various components corresponding to variables that the brain uses to represent the external world:

$$\vec{u}\,\left(t\right)=\left[u_{1}\,\left(t\right),u_{2}\,\left(t\right),\cdots ,u_{N}\,\left(t\right)\right]$$

where the overhead arrow indicates a vector, and *t* indicates that sensory inputs vary (stochastically) over time.

In this paper, we assume that the model used for estimating reward is linear. Although this assumption is common in reinforcement-learning models (Dayan and Abbott, 2001), it is not necessary. We make this assumption here for the sake of simplicity, but address the consequences in the Discussion. The assumption means that a parameter vector:

$$\vec{w}\,\left(t\right)=\left[w_{1}\,\left(t\right),w_{2}\,\left(t\right),\cdots ,w_{N}\,\left(t\right)\right]$$

exists such that the estimated reward is:

(1) $$v\,\left(t\right)=m\,\left(t\right)\,\vec{w}\,\left(t\right)\cdot \vec{u}\,\left(t\right)$$

where 0≤*m*(*t*)≤1 is the motivation function. This equation is important, because learning occurs in the presence of actual rewards by adjusting the *w*’s. The introduction of the m function is a modification of standard reinforcement-learning models, which would use Eq. 1 with *m* = 1. This modification is necessary, since people only get rewards if they act. Thus, if we interpret *m* as the probability of acting, then the received reward is:

(2) $$r\,\left(t\right)=m\,\left(t\right)\,r^{*}\,\left(t\right)$$

where *r*^∗^ is the reward that a fully motivated person would get. The presence of *m* in the reward estimate (Eq. 1) considers that reward itself varies with motivation (Eq. 2).

The typical learning in reinforcement-learning theories follows the precept of temporal difference (Dayan and Abbott, 2001)

(3) δ⁢(t)=r⁢(t)-v⁢(t)

(4) $$\frac{d\,\vec{w}\,\left(t\right)}{d\,t}=k\,\delta \,\left(t\right)\,\vec{u}\,\left(t\right)$$

where *k > 0* is a constant. Equation 4 is a continuous version of the delta rule and thus, tends to minimize the difference between *v* and *r*. Therefore, this minimization makes the estimated reward as close as possible to the real one. An important property of this equation is that because motivation affects both *v* and *r* (Eqs. 1 and 2), it affects learning through the delta (Eq. 3). This is important, since it shows that with no motivation, learning freezes. The freezing makes sense, since for example, if a person is not hungry, then the person will not eat. Thus, the person cannot learn if the estimated reward is large or small in regards to that specific food.

To complete the theoretical framework, we need to specify the statistical properties of $\vec{u}$, *m*, and *r*^∗^ Let us begin by considering $\vec{u}$ and *r*^∗^. Both these variables are exteroceptive signals but with different origins. While $\vec{u}$ is sensory (for example, seeing and smelling an apple) and used for estimating reward, *r*^∗^ arises from the action (for example, eating the apple). As explained in section “General Description of the Theoretical Framework,” these variables are dependent, possibly exhibiting correlation. In a sense, the model acquires this correlation, using it to predict *r*^∗^ from $\vec{u}$. Thus, we are interested in the probability density functions:

(5) $$P\,\left({\vec{I}}_{u}|\vec{B}\right),P\,\left(\left(\vec{u}\,\left(t\right),r^{*}\,\left(t\right)\right)|{\vec{I}}_{u}\right)$$

where $\vec{B}$ indicates the vector of parameters characteristic of the social and environmental background under consideration and ${\vec{I}}_{u}$ is the vector of parameters of an individual in this society. Consequently, the first probability density function in Eq. 5 is the probability of finding an individual, while the second gives the rewards and sensory inputs that this individual gets over time.

Finally, we must specify the statistical properties of *m*. Because it represents interoceptive signals related to motivation, *m* depends on each individual. However, motivation also depends on the sensory input $\vec{u}$. If, say, a person is hungry, but the sensory input is not food, then the individual will not have a motivation to act, that is, to eat. But if by changing the gaze, the sensory input is changed to an appetizing food, the person will be motivated to act. We thus write the probability density function of *m* as:

(6) $$P\,\left({\vec{I}}_{m}|\vec{B}\right),P\,\left(\left(m\,\left(t\right)\right)|\vec{u}\,\left(t\right),{\vec{I}}_{m}\right)$$

where we insert $\vec{B}$ to indicate that individual motivation may depend on environmental and social backgrounds. For example, the motivation to smoke is prevalent in some societies but not others (Dechesne et al., 2013). As for Eq. 5, the first probability density function in Eq. 6 is the probability of finding an individual, while the second gives the motivations that this individual has over time.

## Materials and Methods

We studied the implications of our theoretical framework through mathematical analyses and computer simulations. In section “Methods for Computer Simulations,” we describe the mathematical details of simulating the model, with steps to simplify the procedure. Next, in section “Illustrative Model,” we describe the properties of the illustrative model used in this paper, listing each component and its technical rationale. We then describe the algorithm to simulate the model in section “Summary of the Simulation Procedures.” Finally, we describe the parameters used in the standard simulation in section “Standard Simulation Parameters.” For those readers who do not have a mathematical background, we suggest first reading section “Summary of the Simulation Procedures.” That section may help get an overall understanding before reading the other sections for details.

### Methods for Computer Simulations

We must simulate Eqs. 1–4. Combining these equations, we get:

(7) $$\frac{d\,\vec{w}\,\left(t\right)}{d\,t}=k\,m\,\left(t\right)\,\left(r^{*}\,\left(t\right)-\vec{w}\,\left(t\right)\cdot \vec{u}\,\left(t\right)\right)\,\vec{u}\,\left(t\right)$$

This is a stochastic differential equation, because the $\vec{u}$, *m*, and *r*^∗^ come from samples of the probability distributions in Eqs. 5 and 6.

We simplify our simulations through a mean field approximation of Eq. 6:

(8) $$\frac{d\,\vec{w}\,\left(t\right)}{d\,t}=k\bar{m}\left(\vec{u}\left(t\right):{\vec{I}}_{m}\right)\left(r^{*}\left(t\right)-\vec{w}\left(t\right)\cdot \vec{u}\left(t\right)\right)\vec{u}\left(t\right)$$

where $\bar{m}\left(\vec{u}\left(t\right):{\vec{I}}_{m}\right)$ is the mean motivation as a function of the sensory input $\vec{u}\,\left(t\right)$ and parametric on ${\vec{I}}_{m}$. The advantage of the approximation in Eq. 8 is that we do not simulate the noise in the motivation states, but only their deterministic dependence on the sensory inputs. Nevertheless, the motivation will remain stochastic, because so are the sensory inputs.

To approximate a solution to Eq. 8, we must discretize time and sample $\vec{u}$, *m*, and *r*^∗^ for every *t*. We do this discretization as follows:

(9) $$\vec{w}\left(t_{k+1}\right)=\vec{w}\left(t_{k}\right)+\epsilon \bar{m}\left(\vec{u}\left(t_{k+1}\right):{\vec{I}}_{m}\right)\left(r^{*}\,\left(t_{k+1}\right)-\vec{w}\,\left(t_{k}\right)\cdot \vec{u}\,\left(t_{k+1}\right)\right)\,\vec{u}\,\left(t_{k+1}\right)$$

where ϵ = *k*(*t*_*k* + 1_−*t*_*k*_), with *t*_*k* + 1_−*t*_*k*_ being constant (for k = 0, 1, 2, …).

### Illustrative Model

We performed computer simulations using an illustrative model developed from our theoretical framework. Although this model is just illustrative, we point out in the Results outcomes of mathematical analyses showing that the most important conclusions of the model simulations are general. We also address the generality of the simulation results in the Discussion. In this section, we specify the illustrative model used in the simulations. Because this section is highly technical, we provide a summary of the model with figures in the next section (section “Summary of the Simulation Procedures”). Section “Standard Simulation Parameters” describes the standard parameter set used in the model simulations.

To specify a model, we need to provide the probability functions in Eq. 5, the $P\,\left({\vec{I}}_{m}|\vec{B}\right)$ function in Eq. 6, and the $\bar{m}$ function in Eq. 8. To begin, we took five steps to simplify the model to make the simulations fast:

A. We did not simulate social noise by implementing explicitly $P\,\left({\vec{I}}_{u}|\vec{B}\right)$ and $P\,\left({\vec{I}}_{m}|\vec{B}\right)$. Instead, we set individual parameters by hand, changing them for different individuals to study the parametric dependence of the model:
B. We split the individual parameters ${\vec{I}}_{u}$ into sensory related $\left({\vec{I}}_{s}\right)$ and reward related $\left({\vec{I}}_{r}\right)$:

(10) $${\vec{I}}_{u}=\left[{\vec{I}}_{s},{\vec{I}}_{r}\right]$$

Thus, we divided the ${\vec{I}}_{u}$ parameters into lower-dimensional ones that separately control the samplings of $\vec{u}$ and *r*^∗^.
C. We made $\vec{u}$ two-dimensional. One component was visual balance (*u*_*b*_) and the other was visual complexity (*u*_*c*_), making:

$$\vec{u}=\left[u_{b},u_{c}\right]$$

where 0≤*u*_*b*_, *u*_*c*_≤1, as per the definitions in (Aleem et al., 2017).
  While our model is amenable to a range of sensory inputs, we simplified it to two variables in the visual domain for illustrative purposes. We briefly describe these two variables here, but refer the reader to existing literature to gain a deeper understanding. The component of visual balance but can best be surmised as an equal amount of visual weight across an image, often measured by pixel intensities (Wilson and Chatterjee, 2005). Visual complexity, on the other hand, can be best described as the amount of information in an image, for example the range of pixel intensities present (Donderi, 2006). An important interaction exists between these two variables in that they are generally negatively correlated. For example, as an image becomes more balanced (organized), its complexity generally decreases (Aleem et al., 2017). We explore this relationship in our experiments to test whether the two variables compete and influence learning.
D. We split the second term of Eq. 5 into:

(11) $$P\,\left(\left(\vec{u},r^{*}\right)|{\vec{I}}_{u}\right)=P\,\left(u_{b},u_{c}|{\vec{I}}_{s}\right)\,P\,\left(r^{*}|u_{b},u_{c},{\vec{I}}_{r}\right)$$

Thus, instead of sampling directly from a relatively complex three-dimensional space (two for $\vec{u}$ and one for *r*^∗^), we split the problem. We first sampled from a simpler two-dimensional space and then used the outcome to condition the sampling of a one-dimensional space. The splitting of probabilities in these steps greatly reduces the computation time required for sampling. However, having separate sensory and reward parameters in the two probability distributions increases the degrees of freedom, potentially leading to a wider range of observable behaviors. For example, the sensory and reward functions could be varied independently.
E. To model the various variables in our simulations, we assumed they had Gaussian distributions. While natural scene statistics and neural-reward related processes can have a multitude of probability distributions (Field, 1994), one can often approximate them with Gaussian processes (Wainwright and Simoncelli, 2000; Dabney et al., 2020). Hence, using Gaussian distributions here allowed us to explore the theory from a parsimonious viewpoint. In addition, sampling Gaussian distributions is fast, because of the abundance of code available for this purpose. However, future iterations could benefit from employing other distributions.

We modeled the first term of the right-hand side of Eq. 11 with a truncated bivariate Gaussian distribution (Rosenbaum, 1961),

(12) $$P\left(u_{b},u_{c}|{\vec{I}}_{s}\right)=Tr\left(G_{2}\left(u_{b},u_{c}:\mu_{b},\mu_{c},\Sigma \right)\right)$$

where *G*_2_ is the Gaussian over the variables *u*_*b*_ and *u*_*c*_, with means $\vec{\mu }=\left[\mu_{b},\mu_{c}\right]$ and covariance matrix:

$$\Sigma =\left[\begin{array}{cc} \sigma_{u_{b}}^{2}\; & \rho \,\sigma_{u_{b}}\,\sigma_{u_{c}}\\ \rho \,\sigma_{u_{b}}\,\sigma_{u_{c}}\; & \sigma_{u_{c}}^{2} \end{array}\right]$$

where σ_*u_b*_ and are standard deviations in the *u*_*b*_ and *u*_*c*_ directions respectively, and ρ is the correlation between *u*_*b*_ and *u*_*c*_. In turn, the truncation function *Tr(G_2_)* is:

T⁢r⁢(G⁢(x,y))=1∫01∫01G2⁢(x,y)⁢dx⁢dy⁢{G2⁢(x,y) i⁢f⁢ 0≤x,y≤10 o⁢t⁢h⁢e⁢r⁢w⁢i⁢s⁢e

With these definitions for the first term of the right-hand side of Eq. 11, the individual sensory-parameter vector is therefore,

(13) $${\vec{I}}_{s}=\left[\mu_{b},\mu_{c},\sigma_{u_{b}},\sigma_{u_{c}},\rho \right]$$

To model rewards associated with the sensory variables, we assumed independent contributions of rewards from balance ($r_{b}^{*}$) and complexity ($r_{c}^{*}$), and then summed these contributions, that is,

(14) $$r^{*}=r_{b}^{*}+r_{c}^{*}$$

Hence, if we have $P\,\left(r_{b}^{*}|u_{b},{\vec{I}}_{r}\right)$ and $P\,\left(r_{c}^{*}|u_{c},{\vec{I}}_{r}\right)$, then we can calculate $P\,\left(r^{*}|u_{b},u_{c},{\vec{I}}_{r}\right)$ as:

$$P\left(r^{*}|u_{b},u_{c},{\vec{I}}_{r}\right)=\int_{-\infty }^{\infty }P\left(r_{b}^{*}|u_{b},{\vec{I}}_{r}\right)P\left(r_{c}^{*}=r^{*}-r_{b}^{*}|u_{c},{\vec{I}}_{r}\right)dr_{b}^{*}$$

Consequently, all that remains to do to specify the second right-hand term of Eq. 11 is to define $P\,\left(r_{b}^{*}|u_{b},{\vec{I}}_{r}\right)$ and $P\,\left(r_{c}^{*}|u_{c},{\vec{I}}_{r}\right)$.

To start with the probability density function $P\,\left(r_{b}^{*}|u_{b},{\vec{I}}_{r}\right)$, balance was positively related to reward (Wilson and Chatterjee, 2005). In the simplest mathematical form, balance and reward would obey a linear relationship. We thus define:

(15) $$P\left(r_{b}^{*}|u_{b},{\vec{I}}_{r}\right)=G_{1}\left(r_{b}^{*}:-\alpha +2\alpha u_{b},\sigma_{r_{b}^{*}}\right)$$

where *G*_*1*_ is the univariate Gaussian distribution over the variable $r_{b}^{*}$, and $\alpha ,\sigma_{r_{b}^{*}}>0$ are parameters. The mean of the Gaussian is −α + 2α*u*_*b*_ and the standard deviation is σ_r_b^*_. The mean is such that the integral of −α + 2α*u*_*b*_ over the range of *u*_*b*_(0≤*u*_*b*_≤1) is zero. Positive and negative rewards occur in equal amounts.

We next define $P\,\left(r_{c}^{*}|u_{c},{\vec{I}}_{r}\right)$. Several studies have shown that the preference for complexity displays an inverted U-curve behavior, that is, people like moderate amounts of complexity more than they do little or much complexity (Berlyne, 1971; Donderi, 2006; Güçlütürk et al., 2016). A simple form for the relationship between reward and complexity is a Gaussian shape. We thus define:

(16) $$P\left(r_{c}^{*}|u_{c},{\vec{I}}_{r}\right)=G_{1}\left(r_{c}^{*}:\phi \left(\beta ,\gamma ,\theta \right)+\beta e^{-\frac{{\left(u_{c}-\gamma \right)}^{2}}{2\,\theta^{2}}},\sigma_{r_{c}^{*}}\right)$$

where *G*_*1*_ is now over the variable $r_{c}^{*}$. The parameters are $\beta ,\sigma_{r_{c}^{*}}>0$, 0≤γ≤1, and θ, while ϕ(β,γ,θ) is a function of them. The integral of ϕ + β*exp*(−(*u*_*c*_−γ)^2^/(2θ^2^)) over the range of *u*_*c*_ (0≤*u*_*c*_≤1) is zero. Because ϕ + β*exp*(−(*u*_*c*_−γ)^2^/(2θ^2^)) is the mean of the Gaussian and σ_r_c^*_ is the standard deviation, we have the same amount of positive and negative rewards.

With the definitions in Eqs. 15 and 16, the individual reward parameter vector is therefore,

(17) $${\vec{I}}_{r}=\left[\alpha ,\sigma_{r_{b}^{*}},\beta ,\gamma ,\theta ,\sigma_{r_{c}^{*}}\right]$$

Finally, we defined the motivation function in Eq. 8, namely, $\bar{m}\left(\vec{u}\left(t\right):{\vec{I}}_{m}\right)$. For the sake of simplicity and illustration, we modeled $\bar{m}$ as independent of *u*_*b*_. As for the dependence on *u*_*c*_, we consider different individuals with different peak preferences in terms of complexity. We also use the Gaussian shape to model this peak:

(18) $$\bar{m}\left(\vec{u}\left(t\right):{\vec{I}}_{m}\right)=m_{m\,i\,n}+\left(m_{m\,a\,x}-m_{m\,i\,n}\right)e^{-\frac{{\left(u_{c}-\mu_{m}\right)}^{2}}{2\,\sigma_{m}^{2}}}$$

where 0≤*m*_*min*_,*m*_*max*_,μ_*m*_≤1 and σ_*m*_ are parameters. The parameters *m*_*min*_ and *m*_*max*_ are the minimal and maximal motivations respectively. In turn, μ_*m*_ is the complexity yielding maximal motivation and σ_*m*_ controls how quickly motivation falls as *u*_*c*_ moves away from μ_*m*_. With Eq. 18, the individual motivation parameter vector is:

(19) $${\vec{I}}_{m}=\left[m_{m\,i\,n},m_{m\,a\,x},\mu_{m},\sigma_{m}\right]$$

### Summary of the Simulation Procedures

The simulations proceed with the following algorithm:

a. Suppose that at time *t*_*k*_ the weights are $\vec{w}\,\left(t_{k}\right)$.
b. Sample sensory inputs, $\vec{\text{u}}\,\left({\text{t}}_{\text{k}+1}\right)=\left[{\text{u}}_{\text{b}}\,\left({\text{t}}_{\text{k}+1}\right),{\text{u}}_{\text{c}}\,\left({\text{t}}_{\text{k}+1}\right)\right]$ from Eq. 12.
c. Sample reward for balance, $r_{b}^{*}\,\left(t_{k+1}\right)$ from Eq. 15.
d. Sample reward for complexity, $r_{c}^{*}\,\left(t_{k+1}\right)$ from Eq. 16.
e. Compute overall reward, *r*^∗^(*t*_*k* + 1_) from Eq. 14.
f. Compute motivation, $\bar{\text{m}}\left(\vec{\text{u}}\left({\text{t}}_{\text{k}+1}\right):{\vec{\text{I}}}_{\text{m}}\right)$ from Eq. 18.
g. Compute updated aesthetic weights, $\vec{w}\,\left(t_{k+1}\right)$ from Eq. 9.
h. Start the process again at Step a, but at time *t*_*k+1*_.

**FIGURE 2:**
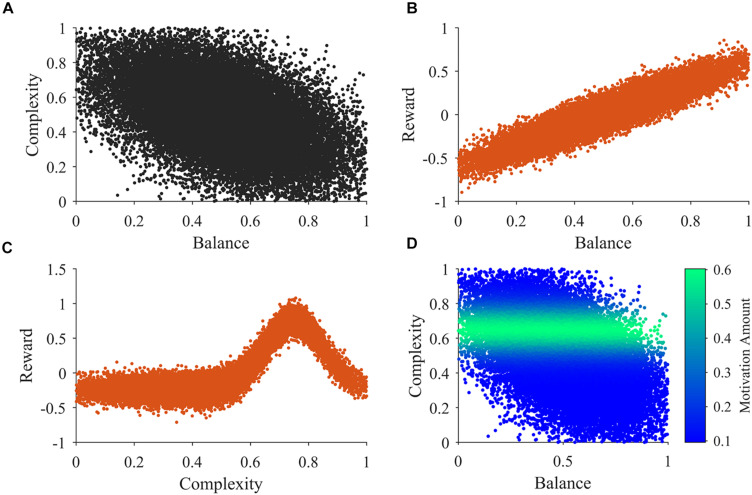
Illustrations of the main functions of the model. **(A)** An illustration of 30,000 samples of the sensory inputs. **(B,C)** Illustrations of observed reward values related to balance and complexity respectively. **(D)** An illustration of the motivation function related to sensory inputs. See Section “Summary of the Simulation Procedures” after Points a-h for a detailed description of these illustrations.

An example of 30,000 samples of the sensory inputs from Step b in a typical simulation appears in Figure 2. Figure 2A illustrates that balance and complexity exhibit negative correlation. Figures 2B,C show typical examples of the distributions used for the samples in steps c and d, respectively. In our model, reward tends to increase linearly with balance, except for the probabilistic distribution of rewards (Wilson and Chatterjee, 2005). Probabilistic fluctuations also affect the dependence of reward on complexity, but the general trend is that of an inverted U-curve behavior (Figure 2C; Donderi, 2006). Importantly, the distributions in Figures 2B,C illustrate that rewards can be both positive and negative. In these figures and in our simulations, positive and negative rewards are balanced, summing to zero. Finally, Figure 2D illustrates the typical shape of the motivation function in Step f. The illustration superimposes color-coded magnitudes of motivation on samples of sensory inputs as in Figure 2A. In our illustrative model, motivation only depends on complexity and has a peak at a particular magnitude of complexity. The peak complexity is distinct for different individuals (not shown in Figure 2D). One may associate individuals with motivations for higher complexity with risk-taking, because high complexity tends to present more uncertainties, at the possible benefit of more information (Furnham and Bunyan, 1988). Similarly, motivations for low complexity may be associated with risk aversion.

All simulations were performed with code specially written in MATLAB R2019b (MathWorks, Natick, Ma, United States). This code is available in an online repository (Supplementary Materials, Section A).

### Standard Simulation Parameters

In this paper, we report on simulations with different parameter sets to explore the model. We have designated one of these sets as our standard set (see Table 1), because the corresponding results capture the data in the literature reasonably well. We also show simulations with other parameter sets to illustrate individual differences and analyze the various behaviors of the model. The table below shows the parameters of the standard simulations. Parameters for other simulations are indicated as appropriate in the Results.

**TABLE 1:** Standard set of parameters.

Parameter(s)	Equation	Values
$\vec{w}\,\left(t_{0}\right)$	9	[0,0]
ϵ	9	0.01
*t*_*k* + 1_−*t*_*k*_	9	1
${\vec{I}}_{s}=\left[\mu_{b},\mu_{c},\sigma_{u_{b}},\sigma_{u_{c}},\rho \right]$	13	[0.5,0.5,0.2,0.2,−0.5]
${\vec{I}}_{r}=\left[\alpha ,\sigma_{r_{b}^{*}},\beta ,\gamma ,\theta ,\sigma_{r_{c}^{*}}\right]$	17	[0.6,0.1,1,0.75,0.1,0.1]
${\vec{I}}_{m}=\left[m_{m\,i\,n},m_{m\,a\,x},\mu_{m},\sigma_{m}\right]$	19	[0.1,0.6,0.65,0.1]

## Results

The following sections outline the results of our simulations and the mathematical analyses. In our first experiments (sections “Learning Dynamics of Aesthetic Weights” and “Understanding the Fast and Slow Phases of Learning”), we looked at the time course of how aesthetic values form by looking at the learned weights. We were particularly interested to see if there were multiple phases (section “Learning Dynamics of Aesthetic Weights”). We found this to be the case, thus in section “Understanding the Fast and Slow Phases of Learning,” we investigated the reasons behind this and found it has to do with the shape of the function linking error between actual and predicted rewards to balance and complexity. The results of our first two experiments also showed that the weights for balance and complexity diverged, indicating an apparent competition. We explored the reasons for this apparent competition by varying different aspects of our model (section “Understanding Apparent Competition between Aesthetic Weights”). We found that motivation was a key component of this apparent competition. Therefore, in the next experiment, we further explored the role of motivation (section “The Role of Motivation and Reward on Aesthetic Individuality”). We found that differing motivation functions can profoundly change the aesthetic weights learned. We then compared this finding to that obtained with differing social reward contingencies and saw a similar effect on aesthetic weights. Finally, we explored the landscape of the learned aesthetic values as a function of complexity and balance (section “Beauty and the Emergence of the Peak-shift Effect”). We discovered that certain regions of the sensory space had higher learned values than average. We hypothesized that this landscape might explain the value-exaggeration effect often observed in art.

### Learning Dynamics of Aesthetic Weights

**FIGURE 3:**
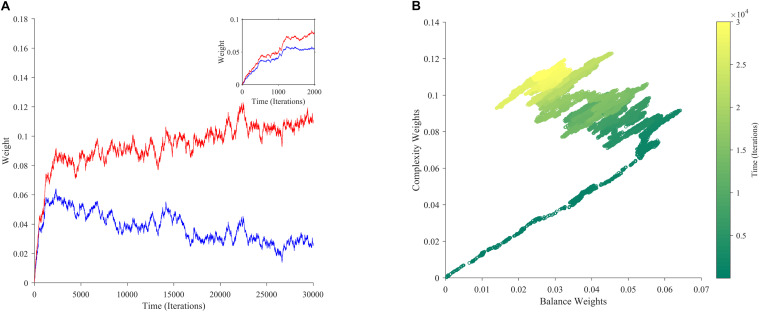
The dynamics of aesthetic weights. **(A)** Balance (blue) and complexity (red) weights as a function of time. The early times of the dynamics appear in the inset. **(B)** A phase-plot of the weights in **(A)** with time color-coded. Initially, the balance and complexity weights grow quickly at similar rates, but later, the complexity weight grows whereas the balance weight falls, both at increasingly slower rates. These simulations of aesthetic-weight dynamics used standard parameters (Table 1).

If aesthetic values are learned, then their corresponding aesthetic weights change over time. Ideally, their dynamics would be so that the values, i.e., the predicted rewards would approach the actual rewards as much as possible. However, weights are not free to change arbitrarily. They may exhibit interdependencies (e.g., Figure 2A), and have different dependences on rewards and motivations (Figures 2B–D). We performed multiple computer simulations to gain an understanding of the dynamics of aesthetic weights. An example with the model described in section “Illustrative Model” and the standard parameters (Table 1) appears in Figure 3.

The simulations in Figure 3A show an example of the dynamics of the aesthetic weights for balance and complexity. The weights start at [0,0], i.e., they reflect a hypothetical individual who knows nothing about the importance of balance and complexity at the initial point of learning (see section “Discussion”). These weights then rise quickly in an initial fast phase and then slow down in a divergent phase. In the initial phase, both balance and complexity weights rise equally in relation to each other (Figure 3A inset). However, after this phase, an inflection point occurs. In the new phase, the complexity weight continues to rise while the balance weight drops, as if they are competing. Thus, these weights reach a state of slow divergence. As time increases, both weights appear to arrive to a stochastic equilibrium in relation to each other, with their separation increasing at a slow pace.

A phase-phase plot is especially helpful to visualize the learning dynamics (Figure 3B). Such a plot graphs the complexity weight as a function of the balance weight, color-coding for time. As the inset of Figure 3A shows, the rise of balance and complexity in the initial phase is tightly correlated, indicated by the linear slope in the phase plot. However, after the inflection point, a much slower drift can be seen through the formation of a cloud region. The dynamic moves slowly toward greater complexity and lower balance, eventually forming a relatively stable stochastic cloud.

**FIGURE 4:**
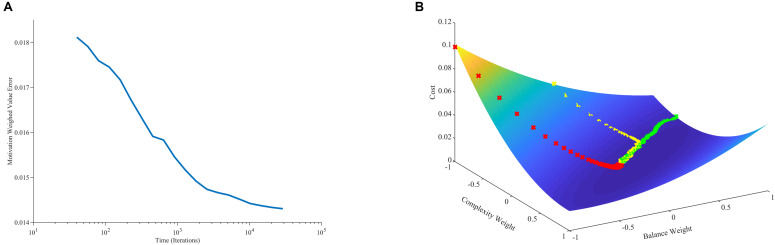
Minimization of error and its relationship to learning dynamics. **(A)** Error between value and reward conditional on the statistics of motivation as a function of time (Eq. 20). **(B)** Error between value and reward as a function of balance and complexity weights with three aesthetic-weight trajectories from different initial conditions. In one trajectory, the error for the initial condition is especially large, leading to a fast, straight descent (red line). In another, the error for the initial condition is especially small, leading to a slow, straight descent (green line). More typically, the descent is initially fast toward the shallow bed, and then curves and slows down when there. These simulations used the standard parameters except for the initial conditions, which were [−1, −1] (red line), [1, −0.6] (green line), and [0, 1] (yellow line).

Why does this stable cloud form in the phase plot? A simple hypothesis would be that the weights gravitate around a fixed point, not converging to it just because of the stochastic nature of our model. Our mathematical analyses show a more complex and interesting picture on the outcome of learning than this hypothesis suggests. The learning process leads to a gradient-descent-like optimization of the prediction of reward (Supplementary Materials, Section B). Specifically, value approaches reward as much as statistically possible as follows: If for every τ there is a *t* > τ such that *m*(*t*) > 0, then the learning process minimizes:

(20) $$E\,\left(\vec{w}\right)={\left\langle \frac{{\left(r\,\left(t\right)-v\,\left(t\right)\right)}^{2}}{m\,\left(t\right)}\right\rangle }_{t}$$

where ⟨ ⟩_*t*_ stands for time average. The minimization of $E\,\left(\vec{w}\right)$ with respect to the components of $\vec{w}$ in Eq. 20 implies that *v*(*t*) tends to become statistically close to *r*(*t*). The near optimization of value in terms of estimating reward as predicted by Eq. 20 is confirmed by our computer simulations (Figure 4A). However, *v*(*t*) does not converge exactly to *r*(*t*) because of two reasons: First, the theoretical framework is stochastic. If the process were not stochastic, then the value would converge exactly to the reward. Second, the optimization of Eq. 20 is modulated by the statistics of *m*(*t*), $\vec{u}$, and *r*^∗^.

However, the mathematical analysis also shows that although value tends to gravitate around a fixed point, the weights do not necessarily do so (Supplementary Materials, Section C). Different sets of weights can produce the same value. To be more precise, we can define the following hyperplane in terms of weights:

(21) $$\sum_{i=1}^{N}a_{i}\,\left(t\right)\,w_{i}\,\left(t\right)=v\,\left(t\right)$$

where they *a*_*i*_(*t*) are:

(22) $$a_{i}\,\left(t\right)=m\,\left(t\right)\,u_{i}\,\left(t\right)$$

such any point in this hyperplane is compatible with the value *v*(*t*). Because of this redundancy, the exact $\vec{w}\,\left(t\right)$ are not always meaningful. Is having such a redundancy in weight representation wasteful? The mathematical analysis shows that this redundancy in weights is not arbitrary, but allows the improvement of the learning rate (Supplementary Materials, Section C). Mathematically, the weights $\vec{w}\,\left(t\right)$ aim to reach the nearest point of the ideal hyperplane in a way that is dependent on their initial conditions. Consequently, because of the stochastic nature of the theoretical framework, the $\vec{w}\,\left(t\right)$ can drift even if the value stays close to reward (Supplementary Materials, Section C). With each new sample of $\vec{u}\,\left(t\right)$, *m*(*t*), and *r*^∗^(*t*), the $\vec{w}\,\left(t\right)$ simply pushes value toward the new hyperplane defined by this sample. Thus, $\vec{w}\,\left(t\right)$ may not return to past positions, possibly drifting according to a random-walk-like trajectory.

### Understanding the Fast and Slow Phases of Learning

What are the underlying reasons for the fast and slow phases of learning observed in Figure 3? Considering that values follow a gradient descent (section “Learning Dynamics of Aesthetic Weights”), we look toward the error between value and reward for an answer. As Figure 4B shows, the error function has a hammock-like shape when plotted against balance and complexity weights. Consequently, the error function varies rapidly along one direction and slowly along its perpendicular. This shape leads to differences in gradients across regions of the function. Thus, if the aesthetic weights start at a point with an especially large error, they will experience a large gradient, descending fast toward the minimum of the function (red line in Figure 4B). If instead they start at a point with an especially small error, they will descend slowly toward the minimum (green line). Ultimately, as the weights approach the minimum of the error function, gradients get smaller and the convergence becomes more stochastic. Thus, weights become more sensitive to variations of sensory inputs, rewards, and motivations. When gradients are steep, weights tend to move to reduce the error rapidly even in presence of input variations. Such initial fast approach is consistent with the fast learning-rate explained in section “Learning Dynamics of Aesthetic Weights.” Hence, the initial conditions may dictate an initial fast approach to the shallow bed, and a more slowly stochastic dependence once there. The direction of gradient descent in the shallow bed is typically different from in the initial fast phase. These different directions lead to curved trajectories (yellow line). Such curved trajectories explain the complex shape of the phase plot (Figure 3B).

### Understanding Apparent Competition Between Aesthetic Weights

**FIGURE 5:**
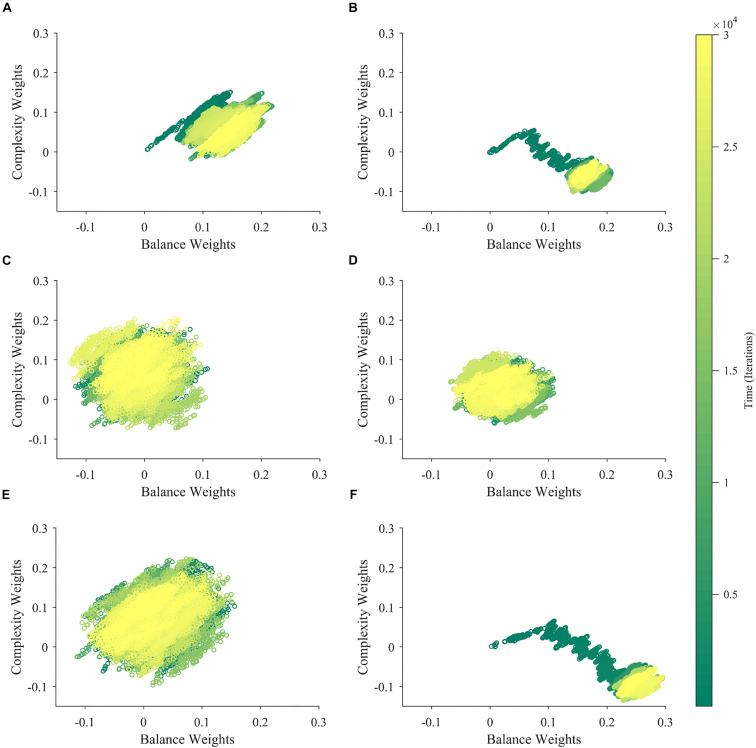
Contributions of the Properties of $\vec{u}$, r^∗^, and m to the Apparent Competition between Aesthetic Weights. **(A)** Eliminating the negative correlation between the components of $\vec{u}$ weakens but does not kill the apparent competition. **(B)** Making the reward function r^∗^ similar for balance and complexity may even strengthen apparent competition. **(C)** Making m a constant leads to the virtual elimination of apparent competition. **(D)** When both m is a constant, and the reward function r^∗^ is similar for balance and complexity, there is no apparent competition. **(E)** The same happens when both m is a constant and we eliminate the negative correlation between the components of $\vec{u}$. **(F)** In contrast, apparent competition remains and can even become stronger when both we eliminate the negative correlation between the components of $\vec{u}$ and the reward function r^∗^ is similar for balance and complexity. Thus, the main factor determining apparent competition in our illustrative model may be the shape of the motivation function.

What is the reason for the apparent competition between balance and complexity weights during the slow phase of learning in Figure 3? A simple hypothesis is that the apparent competition arises from the negative correlation between balance and complexity, i.e., between the components of $\vec{u}$ (Figure 2A). However, inspection of Eq. 7, suggests alternate hypotheses beyond the negative correlation between balance and complexity. For example, they have different reward structures (Figures 2B,C), possibly leading the weight of one becoming more relevant than the other is. Finally, because motivation affects balance and complexity in different manners, it too, could create an apparent competition (Figure 2D). To test these hypotheses, we ran six new simulations varying input correlation, reward structures, and motivation functions. These simulations eliminated the negative correlation between balance and complexity, made the reward structures identical, or set the motivation to a constant independent of balance and complexity. The results of these simulations appear in Figure 5.

When we eliminated the negative correlation between balance and complexity (standard parameters, except that ρ = 0), the apparent competition between their weights did not vanish (Figure 5A). Consequently, this negative correlation is not a necessary condition for the apparent competition. However, the negative correlation affects the apparent competition, because it becomes weaker when we eliminate this correlation, and we see slightly larger balance than complexity weights. Similarly, having different reward structures is not a necessary condition for the apparent competition. If we make the reward structures for balance and complexity identical (both linear as in Figure 2B), the apparent competition remains (Figure 5B). This change leads to an initial rise in both weights followed by an overwhelming relative increase in the balance weight. Finally, in Figure 5C, we remove the effect of motivation from the simulation, by setting *m*≡1. This change results in an isotropic cloud, showing that the shape of the motivation function is a major contributor to the apparent competition. An appropriate motivation function may even be a necessary condition for the apparent competition.

Can the negative correlation between balance and complexity, different reward structures, or the shape of the motivation function be a sufficient condition for the apparent competition between the aesthetic weights? To answer this question, we eliminated two of these conditions at a time. We thus left only one condition in place in each simulation. As seen in Figure 5D, when *m*≡1, and the reward structures are similar for balance and complexity, there is no apparent competition between the weights. Hence, the apparent competition vanishes although the negative correlation is still present. Similarly, when *m*≡1 and we eliminate the negative correlation between balance and complexity, the apparent competition vanishes. It disappears although we still have differences in reward structures (Figure 5E). Thus, neither the negative correlation nor the difference in reward structures is a sufficient condition for the apparent competition. In contrast, the apparent competition continues when we eliminate the correlation and the difference in reward structure, leaving the shape of the motivation function intact. This result thus gives further evidence that the appropriateness of this shape may be a sufficient condition for the apparent competition.

Overall, the key factor for the apparent competition between aesthetic values in our illustrative model may be the motivation function. It generates the apparent competition by modulating both sensory sampling and reward. Negative correlations between the components of the sensory inputs do play a role in the apparent competition but a lesser one.

### The Role of Motivation and Reward on Aesthetic Individuality

**FIGURE 6:**
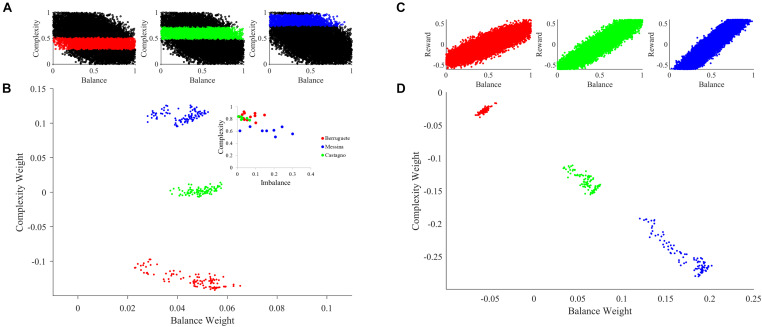
Aesthetic individuality in the theoretical framework. **(A)** Sampling of the motivation function with peak complexities μ_*m*_ = 0.4 (Red), μ_*m*_ = 0.6 (Green), and μ_*m*_ = 0.85 (Blue). **(B)** Sampling of aesthetic weights for simulations with standard parameters except for variations of the peak complexity in the motivation function as color-coded in **(A)**. The inset is the data from Aleem et al. (2017). **(C)** Sampling of the reward as a function of balance with slopes α = 0.5 (Red), α = 1.25 (Green), and α = 1.7 (Blue). **(D)** Same as in **(B)**, except that the slope of the reward as a function of balance varies as color-coded in **(C)**. The sampling started at *t*_*inital*_ = 10,000, was at every Δ*t* = 200, and ended at *t*_*final*_ = 30,000. Variations of either individual parameters such as peak complexity of the motivation function or social parameters such as the slope of the reward-balance function give rise to individuals with different aesthetic values. The expression of individuality is like the data from our previous work (Aleem et al., 2017).

An important consequence of our theoretical framework is that different individuals develop distinct aesthetic weights. If two individuals were from different societies or cultures, they would tend to have differences in their learning parameters. These differences are illustrated by the blue boxes Society 1 and 2 in Figure 1. Mathematically, these individuals would have different *B* parameters in Eqs. 5 and 6. However, even if individuals came from the same society, their learning parameters would tend to be distinct (blue boxes labeled Individual 1 and 2 in Figure 1). Again, in Eqs. 5 and 6, these individuals would have different ${\vec{I}}_{u}$ and ${\vec{I}}_{m}$ parameters. In Figure 6, we illustrate through computer simulations how this individuality emerges.

To illustrate the effect of individualized learning of aesthetic value in a society, we modeled a scenario for the case of motivation for complexity. While we could have investigated motivation for balance as well, existing research shows different personality traits can account for changes in preference for complexity (Furnham and Bunyan, 1988). Furthermore, it has been shown that the motivation for complexity can be experimentally manipulated (Tinio and Leder, 2009). Thus, we sought to see what happens when motivation for complexity changes across individuals. To test this hypothesis, we varied the peak complexity of the motivation function (Figures 2D, 6A). The larger the peak complexity is, the more motivation the individual must act on high complexity. As seen in Figure 6B, changing this form of motivation has a direct effect on learned aesthetic weights. Specifically, when the motivation is shifted toward high complexities, late aesthetic weights weight for balance becomes weaker. In contrast, those for complexity become stronger. Hence, the three different individuals in Figure 6B (in terms of peak complexity) express different learned aesthetic weights. Moreover, because our theoretical framework is stochastic, the aesthetic weights form clouds in limited regions of the so-called neuroaesthetic space (Aleem et al., 2017). The separation and partial overlap between these clouds is like what we observe for different artists (inset of Figure 6B).

In turn, to illustrate how social variations may influence aesthetic learning of individuals, we modulated the reward structure for balance (Figures 2B, 6C). The larger the slope of this structure, the more social reward an individual got with highly balanced sensory inputs. As a result, we can see in the Figure 6D that the changing of the reward structure has an appropriate effect on aesthetic weights. Increasing the balance slope expectedly increases the weights toward balance, reducing those for complexity. Again, the three different individuals in Figure 6D (in terms of social reward structure) express different learned aesthetic weights. Finally, once again, our theoretical framework is stochastic. Consequently, the aesthetic weights form clouds in limited regions of the neuroaesthetic space as also seen in the analysis of aesthetic weights in portraits by master painters of the Early Renaissance (Aleem et al., 2017).

In conclusion, individuality in aesthetic learning emerges in the theoretical framework through variations in either cultural norms or individual motivational states.

### Beauty and the Emergence of the Peak-Shift Effect

Any theory for aesthetic learning must account after convergence for as many relevant properties in the literature as possible. So far, we have discussed the dynamics of learning aesthetic weights. However, we have not yet explored the amount of value possible in different regions of the neuroaesthetic space. This exploration naturally leads us to the broader question of creation of art and beauty. Where does beauty, or in our case, regions of highest value, exist? We looked to the literature for existing hypotheses on this question. One of the most prominent hypotheses in this regard has to do with the “peak-shift” effect (Ramachandran and Hirstein, 1999). It supposes that the beauty of an object is partly owed to the exaggeration of some of its characteristics. According to the hypothesis, if an attribute signals value normally, exaggerating that attribute would lead to greater value. This effect is theorized to explain the tendency of artists to exaggerate variables that contribute to aesthetic emotions. Accordingly, visual artists should tend to exaggerate certain statistical properties like symmetry, complexity or even certain facial features as compared to what one observes typically (Costa and Corazza, 2006; Graham and Redies, 2010; Aleem et al., 2017). Thus, because of this exaggeration effect, beauty is not merely copying reality.

**FIGURE 7:**
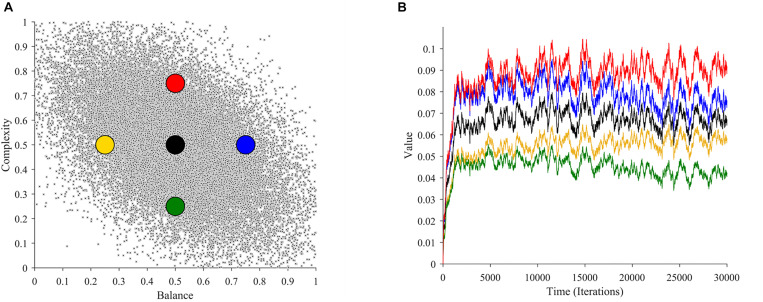
Illustration of the Value-exaggeration effect as an emergent property. **(A)** The distribution of balance and complexity (as in Figure 2A) with overlaid points from which we calculate value. **(B)** Aesthetic values as a function of time at the overlaid points in **(A)** (with the same color-code). The black dot represents the most typical (most probable) images. The yellow and red images represent stimuli regions with the least value, whereas the blue and green discs represent regions with the most value. Therefore, to increase the aesthetic liking, artists should strive to paint in the red and blue regions, or more generally, in the upper-right quadrant.

In this section, we study whether and why our theoretical framework is consistent with such a peak-shift effect. To perform this study, we calculated aesthetic values of images with different complexities and balances in our simulations. We used these calculations to compare the aesthetic values of images with the most typical statistics with those with less probability of occurring. The comparison appears in Figure 7.

As seen in Figure 7A, there are many possible regions of the neuroaesthetic space that a certain scene or painting could occupy. However, not all these regions are identical in terms of value. For example, the region indicated by the black dot represents what is most typical. If we turn to Figure 7B, we see that this region leads to the learning of moderate overall value. It is apparent that there are regions with greater or lesser value. In our example in Figure 7A, the yellow and green dots represent regions with lower value in relation to the black dot. In turn, the regions around the red and blue dots yield greater value than the regions around the black dot. Consequently, if an artist wants to maximize value, they would be keen to paint with attributes in the regions of the upper-right quadrant of this neuroaesthetic space. In our case, artists would thus tend to exaggerate the complexity and balance concurrently to increase the aesthetic appeal of their work.

Mathematical analyses show that this value-exaggeration result is a general property for our theoretical framework (Supplementary Materials, Section D). Thus, this result is applicable beyond the parameters of the simulations in Figure 7. The analysis also extends the results for broad classes of learning models that are nonlinear, that is, not following Eq. 1. If the properties of these learning models and of the probability distribution of sensory signals obey general conditions, then the value-exaggeration result will hold. The linear model in Eq. 4 will almost always obey these conditions.

In conclusion, our analyses support the peak-shift effect. Our framework does so by predicting that the most typical inputs are not necessarily the sources of highest predicted value. Other possible inputs yield more aesthetic value than reality, thus appearing more beautiful. As an important extension, our theoretical framework predicts that the value landscape of possible inputs is different for each individual. Our explanation for this effect is thus that what matters for aesthetics is not the statistics of sensory inputs but their values to perceivers.

## Discussion

The field of neuroaesthetics has progressed rapidly lately, especially with regards to the understanding of the “what” and “where” of aesthetic preferences. However, one of the biggest remaining questions has to do with “how” we develop aesthetic values in the first place (Göksun et al., 2014). The origin of certain preferences such as that for contour, symmetry, or contrast can be explained by evolutionary accounts (Reber et al., 2004; Bar and Neta, 2006). However, even these seemingly innate preferences are subject to experience dependent changes (Germine et al., 2015; Huang et al., 2018). Therefore, it is important to consider the dynamics of aesthetic preferences over time. Here, we presented a theoretical contribution to the understanding of these dynamics. Our theoretical framework proposes that learning forms a large component of aesthetic values. We operationalized this proposal through a computational model of reinforcement learning. We discuss our interpretation of some of the important findings of this modeling effort in greater detail here. For a quick summary of the results, please refer to the beginning of the Results section.

### Interpretations of the Simulation Results

Our results suggest that the time course of aesthetic learning has different phases. Such a multi-phase result is expected from a reward-based learning model. If complete prior inexperience is assumed, then a rapid initial phase is bound to happen, followed by a slower one. These fast-then-slow learning dynamics are characteristic of reinforcement-learning frameworks, where the change of weights is proportional to the error (Dayan and Abbott, 2001; Sutton and Barto, 2018). The fast phase corresponds to the large initial errors, but when they decrease, the learning rate slows down. Our decision for setting the initial weights at zero allows us to understand general principles of learning and to see that it has distinct phases. Such fast aesthetic learning should occur mostly early on in life. However, whether we are born without any aesthetic preconceptions is an open question. What is the likelihood that we come across a completely novel stimuli in our adult lives? More likely, we see previously learned attributes in a novel way, as for example, a drummer seeing a table for the first time. Or take abstract art, which combines familiar visual primitives in a novel way. In these cases, some type of initial prediction of value may exist, albeit with low confidence and high noise. Here too, our theoretical framework would predict that as soon as learning occurs it would be multi-phasic, rapid initially, and then coming to a consolidation.

A surprising result of our model was the apparent competition between aesthetic variables in the second phase of learning. We investigated the source of this apparent competition in our illustrative model and found that motivation is the main driving force. Therefore, motivation appears to be crucial in guiding different trajectories of learning. While motivation in our model seems like a simple gating mechanism, the results show that motivation has complex consequences on learning. This is due to the probabilistic dependence of motivation on sensory inputs and social rewards. Hence, motivation allows for state-dependent learning, accounting for aesthetic diversity and individuality. This result makes intuitive sense, as while we may all start from similar points, our motivations to engage with certain objects and environments will be different. Therefore, different motivations will lead us to divergent paths. These motivations in turn will be influenced by many internal and external factors including the environment and social standards. The interaction and co-dependence of these factors leads to many unique outcomes from a shared starting point.

A unique aspect of our theoretical framework and thus, of our illustrative model is their statistical nature. Coupled with the nonlinearities in the model, this statistical nature leads to many important and surprising results. One such result is the apparent competitive interactions mentioned above. Another important result is that the learned aesthetic weights are stochastic, that is, we should not expect them to be constant and stable, but to fluctuate over time. In our illustrative examples with only two variables, the aesthetic values eventually converge stochastically around a fixed point. The situation should be more complex in the real world, because the number of variables would be higher, possibly leading to multiple fixed points instead of just one. Such a multiplicity would give the appearance of multiple possible aesthetic stable states. Another complexity stems from the key difference between aesthetic weights and values. Aesthetic weights are different from the values, as different weights can lead to same values. Therefore, any apparent fixed-point in aesthetic weights could drift over time adding more variability to aesthetic preferences. The associated aesthetic values, as seen in Figure 7 are stochastic as well. Therefore, our theoretical framework makes a surprising prediction that aesthetic preferences are not the same from one moment to another. This goes against the common assumption that our preferences are relatively stable and thus, that we only need to account for them once. In support of this, converging evidence is beginning to call the long-held assumption of preference stability into question (Höfel and Jacobsen, 2003; Chen and Risen, 2010; Kościński, 2010; McManus et al., 2010; Pugach et al., 2017). The observed instability is commonly attributed to noise within the internal sampling of subjective values. Our theoretical framework makes an additional prediction that the values themselves may be stochastic.

### Limitations and Outlook

At first glance, our theoretical framework for aesthetic learning may seem too reductionist. Aesthetic experiences are complex and many factors are at play. We propose that low-level features and reward-based learning forms just one component of acquiring and using aesthetic values. Our theoretical framework does not address other important aspects for aesthetic emotions, such as semantics, attention, and memory (Leder et al., 2004). We acknowledge these factors play a role in the formation of aesthetic values and their omission is not to undermine them. Instead, we chose to limit the complexity of our theoretical framework at this first iteration to serve as a basic building block on which to incorporate the aforementioned factors. However, even a model based on low-level features can still be highly informative on aesthetic preferences of individuals, as recently demonstrated by Iigaya et al. (2020). Additionally, a reinforcement learning circuit is easily amenable to additional factors, for example Leong et al. incorporate attention directly into the reinforcement-learning circuitry computing subjective value, as we did with motivation (Leong et al., 2017).

A factor that features prominently in studies of aesthetic preference formation but not considered in our framework here is familiarity (Zajonc, 1968). Numerous studies show that increased familiarity tends to improve appraisal, with no apparent reward (Leder et al., 2004; Park et al., 2010; Lindell and Mueller, 2011). However, we argue that familiarity may either be intrinsically rewarding or a promoter to value. For instance, every new exposure leading to familiarity improves our processing of the stimuli. This improvement likely facilitates object recognition and ability to extract semantic and emotional content, which is rewarding (Reber et al., 2004). At a conceptual level, familiarity is intimately tied with novelty, which may also be intrinsically rewarding (Biederman and Vessel, 2006). However, the relationship between these two factors is not entirely clear. For example, roles of familiarity and novelty may be dependent on context (Tinio and Leder, 2009; Park et al., 2010). To help understand this, future iterations of our framework would benefit by incorporating these related factors. For example, one could incorporate an aspect of reward that is contingent both on the novelty and the number of exposures to certain stimuli, as suggested by Biederman and Vessel (2006). As of now, we do not differentiate between these aspects of reward, but this distinction is important and a necessary addition for the future.

As far as implementation, our model assumes a linear relationship between aesthetic weights and values (Eq. 1). Biologically, this linearity is not reflective of typical reward-related synapses (Schultz, 2015). Recent assessments of the field of neuroaesthetics have signaled the need for a new conception of aesthetics that incorporates distributed processing and non-linear recurrent networks (Leder and Nadal, 2014; Nadal and Chatterjee, 2019). While we agree, we suggest that linearity is a suitable starting point. Recent work comparing a linear rule versus a deep neural network to predict subjective aesthetic value found that both fared comparably (Iigaya et al., 2020). We argue that this also applies for our theoretical framework for aesthetic learning. We have shown that most nonlinearities can explain the value-exaggeration effect (section “Beauty and the Emergence of the Peak-shift Effect” and Section D of Supplementary Materials). Most of our other results would likely be similar if we used a monotonic nonlinear relationship between aesthetic weights and values. For example, Eq. 4 is all that we need to explain the fast-then-slow dynamics of aesthetic learning. From that equation, when the error is large or small, so is the rate of learning, regardless of whether the relationship between aesthetic weights and values is linear. Likewise, Eq. 4 is all that we need to explain the near optimality of the learned value, because this equation predicates the tendency of the minimization of error. Consequently, near optimality should occur regardless of whether the relationship between aesthetic weights and values is linear. We can make similar arguments for the non-necessity of linearity for almost all the results in this paper. Thus, assuming a linear relationship between aesthetic weights and values is not a major problem for the validity of our results.

Other limitations are not with the theoretical framework but with the illustrative model. For example, we limit the sensory inputs in our model to two visual statistics. While this simplifies our simulations of the model, it is not reflective of the external world, where a deluge of variables is at play. These variables may all exist in some complicated multi-dimensional space, which we have previously termed the “neuroaesthetic space” (Aleem et al., 2017). Future implementations would certainly have to increase the dimensionality and type of sensory inputs into the model.

Overall, our model, while limited, provide a platform for further research, such as by increasing the richness of the model in the many ways mentioned above. Equally as important are efforts to test empirically the predictions of the model. More developmental and longitudinal empirical studies of aesthetic preferences are needed. For example, one could conduct extensive reinforcement learning studies to determine how learning modulates subjective values over long periods as shown by Wimmer et al. (2018). Similarly, one could empirically test the prediction of temporal variability in aesthetic values.

### Compatibility With Existing Frameworks

Where does our contribution fall into place within the existing theories of aesthetics? Most of the extant theoretical frameworks for aesthetics aim to explain the phenomena at hand. That is not to say that some of these frameworks do not consider the importance of temporal aspects, albeit implicitly. In particular, learning is a key part of many of the existing influential theoretical frameworks. For example, in Chatterjee and Vartanian’s “Aesthetic Triad” model, aspects of learning and reward make two out of the three nodes (Chatterjee and Vartanian, 2014). Others have made reward-based learning central to their theories. For example, in formulating the “Aesthetic Preference Formation” model, Skov defines nodes associated with sensory stimuli, reward prediction, learning, and context amongst others (Skov, 2010). Like us, he stresses the involvement of a reinforcement learning mechanism that is not unique to aesthetics.

In regards to the time frames of learning, Nadal and Chatterjee describe three time-scales influencing aesthetic preferences (Nadal and Chatterjee, 2019). Our model is most like their middle-range time scale, which concerns “individual experience and cultural context.” In a similar vein, Vessel and colleagues build on the reward circuitry with an explicit emphasis on time (Biederman and Vessel, 2006; Vessel and Rubin, 2010). Like us, they implicate associative processes as a central mechanism of time-dependent preference formation. In their view, aesthetic preferences are shaped by the temporal summation of their associative components. In contrast to us, their theory mainly focuses on mechanisms of shared associations. For example, most people will have favorable memories of beaches, therefore leading to a large consensus of preference. However, our model accounts for this preference effect as well by incorporating social statistics of rewards. Another important aspect of Vessel and colleagues work is that “deeper” and more meaningful rewards will lead to stronger preferences. While we do not consider this aspect in our framework, it would be a compatible addition.

Unlike the theories mentioned above, our theoretical framework is specified in a fully computational manner. However, other theories are computational, too! Perhaps the earliest computational theory of aesthetics comes from Martindale. This theory largely focuses on pleasure, formulating that the enjoyment derived from an aesthetic stimulus is proportional to the number of cognitive units activated by it, as envisioned in a neural network (Martindale, 1984). Another computational theory comes from Schmidhuber, who contends that aesthetic preferences are largely driven by intrinsic reward (Schmidhuber, 2010). According to him, when we learn new things and improve our predictions of the world, we maximize this reward. This idea is similar to the theory put forth by Van de Cruys and Wagemans who propose that aesthetic value results from the resolution of prediction errors caused by ambiguities in art (Van de Cruys and Wagemans, 2011). However, all of these theories are primarily focused on the nature of the aesthetic experience and its ensuing reward. While these theories use reinforcement learning circuitry as their basis, unlike us, they do not explicitly consider the learning of aesthetic values over time. But both Schmidhuber and Van de Cruys and Wagemans do argue that experience with different environments over time will lead to differences in predictions, accounting for individual and cultural differences. Nevertheless, unlike these theories, we do not concern ourselves with the nature of reward. In our view, rewards form aesthetic values, whether the rewards are external or internal.

In our framework, the predicted reward, often termed “value” is akin to aesthetic value. This is arguably the most important assumption of our framework. Thus, we propose that our prior experiences with an object influence the values assigned to the many attributes of that object. When we sense these attributes in a new sensory input, their associated values will influence our aesthetic preference for that input (Barrett and Bar, 2009). An illustration of this aesthetic value transfer effect is shown by Strauss et al. (2013). They found that preferences for two-dimensional color patches were systematically altered by looking at positive or negative objects of the same color. More direct studies using instrumental conditioning show that preferences for cues proportionally coincide with their ability to predict reward, even when subliminal (Pessiglione et al., 2008). Therefore, we contend that as certain stimulus attributes signal reward, they themselves become secondary reinforcers, and hence obtain aesthetic value. For example, humans and other animals may have initially preferred symmetry because of its health cues, such as in faces (Treder, 2010). However, after eons of evolution, symmetry may now be a secondary reinforcer itself, signaling value independently (Pombo and Velasco, 2019). Neuroimaging studies support this viewpoint, showing that secondary reinforcers activate similar regions associated with pleasure as do primary reinforcers (Sescousse et al., 2013).

Whether the pleasure from aesthetic value is phenomenologically distinct from other pleasures is an open question (Christensen, 2017; Nadal and Skov, 2018; Menninghaus et al., 2019; Skov and Nadal, 2020). On the one hand, neuroimaging studies show that a range of rewarding, pleasure-giving experiences are processed in the same brain regions. This common processing of reward allows us to make value-based decisions across various goods (Levy and Glimcher, 2012). On the other hand, network-based studies of deeply rewarding phenomena show the concurrent role of other brain processes (van Elk et al., 2019). For example, the default-mode network has been shown to be modulated by intense aesthetic experiences (Vessel et al., 2012). Thus, a subjectively deep experience is likely to activate different brain networks, yet simultaneously be under the constrain of the neurobiological roots of “basic” pleasures. The broader implications of these differences remain to be discovered.

### What Is Beautiful According to Our Theoretical Framework?

“Beauty is natures brag.” Thus, the poet John Milton wrote in praise of the beauty that one often experiences in nature (Milton, 1858). We hear of such experiences commonly, but not all natural scenes are pleasant or breathtaking. For example, some scenes may be repulsive to some people by including a rotting corpse, an approaching snake, or a spider web. What is it that makes some natural scenes beautiful? Following from the discussion in section “Compatibility with Existing Frameworks,” our theoretical framework proposes that when a certain natural scene appears beautiful, it does so, because its statistics elicit high positive value. Our results showed that when looking at the overall value landscape, certain regions that are far away from the norm will correspond to higher value (Figure 7). These are the regions that our theoretical framework may consider “beautiful.” Accordingly, only the minority of scenes might be truly beautiful by eliciting high values. These are the scenes that exaggerate high-value attributes. This sentiment is similar to Ramachandran and Hirstein’s application of the “peak-shift” effect to beauty, proposing that it often comes from exaggeration (Ramachandran and Hirstein, 1999). We thus may ask, to what “exaggerated” natural scenes was Milton referring to? Perhaps, Milton’s scenes would have some sort of exaggerated statistics related to attributes that were innate, that is, formed due to evolutionary pressures (Aleem et al., 2019). Alternatively, according to the theoretical framework here, Milton’s scenes would have exaggerated sensory statistics related with positive experiences in his youth.

Why would the brain evolve a mechanism that prefers exaggeration rather than the most common reality? We argue that any learning system such as the brain would likely prefer exaggeration if its goal is to maximize reward. Consequently, perhaps evolution has allowed our ancestors to choose actions that maximize value. However, there are limits to exaggeration. For example, our results show that exaggeration in the wrong direction will lead to lower than normal aesthetic values, or what one may consider “ugly.”

In sum, our theoretical framework extends the peak-shift hypothesis through individualized value exaggeration. According to the framework, the aesthetic weights that maximize reward are not universal across all individuals. Each person has an individual set of near-optimal aesthetic weights according to personal motivations, and social and environmental contexts. Neuroaesthetic-space regions of high value, or beauty, to one may be regions of low value or ugly to another. We conclude that the different senses of beauty across individuals are not arbitrary, but stem from a personalized near optimization of values.

## Data Availability Statement

The datasets presented in this study can be found in online repositories. The names of the repository/repositories and accession number(s) can be found in the article/ Supplementary Material.

## Author Contributions

HA and NG were involved in developing the framework and writing and revising the manuscript. NG developed the equations and carried out the mathematical analyses. HA carried out the simulations. IC-H helped to edit and revise the manuscript. All authors contributed to the article and approved the submitted version.

## Conflict of Interest

The authors declare that the research was conducted in the absence of any commercial or financial relationships that could be construed as a potential conflict of interest.
