# Supplementary material for: A Theoretical Framework for How We Learn Aesthetic Values
Source: Front Hum Neurosci. 2020 Sep 11;14:345. doi: 10.3389/fnhum.2020.00345 (PMC7518219; doi:10.3389/fnhum.2020.00345)
Supplement: Supplementary file 1 [file Table_1.docx]

Supplementary Materials

# Computer Code

The computer code used in the simulation is available in the online repository at <https://github.com/ha554n/AestheticLearning/blob/master/README.md>

# Optimization of the Learning of Value

*Claim 1*

If for every $\tau$ there is a $t>\tau$ such that $m\left( t \right)>0$, then the learning process minimizes

| (S1) | $E\left( \vec{w} \right)=\left\langle m\left( t \right)\left( r^{*}\left( t \right)-\vec{w}\left( t \right)\cdot\vec{u}\left( t \right) \right)^{2} \right\rangle_{t},$ |
| --- | --- |

where $\left\langle\right\rangle_{t}$ stands for time average.

*Proof*

The gradient of $E$ with respect to the components of $\vec{w}$ is

|  | $\nabla_{w}E\left( \vec{w} \right){\propto-\left\langle m\left( t \right)\left( r^{*}\left( t \right)-\vec{w}\left( t \right)\cdot\vec{u}\left( t \right) \right)\vec{u}\left( t \right) \right\rangle}_{t},$ |
| --- | --- |

|  | $\nabla_{w}E\left( \vec{w} \right)\propto-\left\langle r\left( t \right)-v\left( t \right)\vec{u}\left( t \right) \right\rangle_{t},$ |
| --- | --- |

or

| (S2) | $\nabla_{w}E\left( \vec{w} \right)\propto-\left\langle\delta\left( t \right)\vec{u}\left( t \right) \right\rangle_{t}.$ |
| --- | --- |

Therefore, the process governed by Eq. 4 minimizes $E\left( \vec{w} \right)$ by performing a gradient descent (Strutz, 2010).

*Comments on Claim 1*

- The minimization of $E\left( \vec{w} \right)$ with respect to the components of $\vec{w}$ in Eq. A1 implies that $\vec{w}\left( t \right)\cdot\vec{u}\left( t \right)$ becomes statistically close to $r^{*}\left( t \right)$. Equivalently, $v\left( t \right)=m\left( t \right)\vec{w}\left( t \right)\cdot\vec{u}\left( t \right)$ becomes statistically close to ${r\left( t \right)=m\left( t \right)r}^{*}\left( t \right)$. Hence, the process optimizes value by making it as close as possible to reward.
- However, $v\left( t \right)$ does not typically converge exactly to $r\left( t \right)$. Two reasons may contribute to this “failure” of convergence: 1. Depending on the nonlinearities of the model of reward, the gradient descent may get stuck in local minima. 2. Although Equation S2 implies a minimization of the average of $\delta\left( t \right)\vec{u}\left( t \right)$, the process of change of $\vec{w}\left( t \right)$ depends on a process in which $m$, $\vec{u}$, and $r^{*}$ change stochastically moment by moment. Consequently, correlation between these variables may cause an end result different from that predicted by the minimization of Eq. S1.
- The requirement that for every $\tau$ there is a $t>\tau$ such that $m\left( t \right)>0$ is necessary to give the process enough time to reach optimization. If $m\left( t \right)=0$ for every $t>\tau$, then the learning process freezes after $\tau$ as shown by Eqs. 1-4.

*Claim 2*

If $\vec{u}\neq0$, $m>0$, $r^{*}$ are constant, then

| (S3) | $\lim_{t\to\infty} v\left( t \right)=r.$ |
| --- | --- |

*Proof*:

By combining Eqs. 3 and 4, we get

|  |  | |
| --- | --- | --- |
| (S4) | | $\frac{d\vec{w}\left( t \right)}{dt}=k \left( r-v\left( t \right) \right) \vec{u}$ |

or

| (S5) | $\frac{d\vec{w}\left( t \right)}{dt}=k \left( r-m\vec{w}\left( t \right)\cdot\vec{u} \right) \vec{u}.$ |
| --- | --- |

The existence of a stable fixed point depends on the properties of the second term of the right-hand side of Eq. S5 (Karamardian, 2014). This term can be written as

|  | $-m\left( \vec{w}\left( t \right)\cdot\vec{u} \right)\vec{u}=-m \left( \begin{matrix} \sum_{i=1}^{N} w_{i}\left( t \right)u_{i}u_{1} \\ \vdots\\ \sum_{i=1}^{N} w_{i}\left( t \right)u_{i}u_{N} \end{matrix} \right)$ |
| --- | --- |

or

| (S6) | $-m\left( \vec{w}\left( t \right)\cdot\vec{u} \right)\vec{u}=-m U \vec{w}\left( t \right),$ |
| --- | --- |

where U is the matrix operator

| (S7) | $U=\left( \begin{matrix} u_{1}u_{1} & \cdots& u_{1}u_{N} \\ \vdots& \ddots& \vdots\\ u_{N}u_{1} & \cdots& u_{N}u_{N} \end{matrix} \right).$ |
| --- | --- |

Because the matrix in Eq. S7 is positive definite its eigenvalues are positive (Horn and Johnson, 1990). This shows after the multiplication by the negative $-m$ that the system has a stable fixed point (Moya-Cessa and Soto-Eguibar, 2011). This fixed point occurs when Eq. S4 converges to 0, that is, when $v\left( t \right)$ converges to $r$.

*Comments on Claim 2*:

- Claim 1 showed that $v\left( t \right)$ does not converge exactly to $r\left( t \right)$ in general because of the stochastic nature of the theoretical framework. However, Claim 2 shows that when we eliminate its probabilistic nature, the convergence becomes exact.
- The requirement that $\vec{u}\neq0$ stems from the same reason why we need $m\left( t \right)\neq0$. If either $\vec{u}=0$ or $m\left( t \right)=0$, then the learning process freezes. Thus, an individual behaving as described by our theoretical framework needs relatively strong motivation and “interesting” sensory signals to learn value effectively.

# Non-uniqueness of Weights and Maximization of the Learning Rate

*Claim 3*

An infinite number of $\vec{w}\left( t \right)$ are compatible with any given $v\left( t \right)$.

*Proof*

We can rewrite Eq. 1 as follows

| (S8) | $\sum_{i=1}^{N} a_{i}\left( t \right)w_{i}\left( t \right)=v\left( t \right),$ |
| --- | --- |

where the coefficients $a_{i}\left( t \right)$ are

| (S9) | $a_{i}\left( t \right)=m\left( t \right)u_{i}\left( t \right).$ |
| --- | --- |

The linear polynomial in Eq. S8 describes a time-dependent, N-1-dimensional hyperplane on the weight variables $w_{i}\left( t \right)$. Any point in this hyperplane is compatible with the value $v\left( t \right)$.

*Comment on Claim 3*

- Hence, although value tends to approach reward in a statistically optimally way, this convergence can happen with different sets of weights. Weights themselves do not necessarily converge or gravitate around a stable fixed point.

*Claim 4*

The trajectory of $\vec{w}\left( t \right)$ tends to be the shortest possible towards achieving $v\left( t \right)\to r\left( t \right)$.

*Proof*:

At time t, the $\vec{w}\left( t \right)$ are at point of the weight space are such that the value is $v\left( t \right)=m\left( t \right)\vec{w}\left( t \right)\cdot\vec{u}\left( t \right)$. However, the ideal value should be equal to the reward, that is, $r\left( t \right)=m\left( t \right)\vec{w}_{I}\left( t \right)\cdot\vec{u}\left( t \right)$, where $\vec{w}_{I}\left( t \right)$ are ideal weights. This equation describes a hyperplane over the variables $\vec{w}_{I}\left( t \right)$. All points in this hyperplane have an ideal value, namely, $v_{I}\left( t \right)=r\left( t \right)$. The shortest path from point $\vec{w}\left( t \right)$ to this ideal hyperplane is via a straight line perpendicular to the hyperplane passing through $\vec{w}$. Vectors perpendicular to this hyperplane are parallel to $\vec{u}\left( t \right)$ (Cheney and Kincaid, 2009). Therefore, the shortest path from point $\vec{w}\left( t \right)$ to the hyperplane must move in a direction parallel to $\vec{u}$. That is exactly how Eq. 4 moves the weights $\vec{w}\left( t \right)$.

*Comments on Claim 4*:

- The redundancy of $\vec{w}\left( t \right)$ is not wasteful. It allows learning to push the value as fast as possible towards the reward.
- Because of this redundancy, the exact $\vec{w}\left( t \right)$ not always meaningful. The weights $\vec{w}\left( t \right)$ aim to reach the nearest point of the ideal hyperplane in a way that is dependent on their initial conditions. What prevents this process from being perfect is that the ideal plane changes from moment to moment according to the statistics of $m\left( t \right)$, $\vec{u}$, and $r^{*}$.
- Consequently, because of the stochastic nature of the theoretical framework, the $\vec{w}\left( t \right)$ can drift even if the value stays close to reward. With each new sample of $\vec{u}\left( t \right)$, $m\left( t \right)$, and $r^{*}\left( t \right)$, the $\vec{w}\left( t \right)$ simply pushes value towards the new hyperplane defined by this sample. Thus, $\vec{w}\left( t \right)$ may not return to past positions, possibly drifting according to a random-walk-like trajectory (Durrett, 2010).

# Value Exaggeration

*Claim 5*

There are sensory signals $\vec{u}\left( t \right)$ with better value $v\left( t \right)$ than the mean sensory signal

| (S10) | $\left\langle\vec{u} \right\rangle=\int_{\vec{u}} P\left( \vec{u} \vert\vec{I}_{u} \right)\vec{u} d\vec{u},$ |
| --- | --- |

except in rare degenerate cases of $P\left( \vec{u} | \vec{I}_{u} \right)$ (see Eq. 5 for the definition).

*Proof*

The value for the mean sensory signal $\left\langle\vec{u} \right\rangle$ is

|  | $v_{\left\langle\vec{u} \right\rangle}\left( t \right)= \vec{w}\left( t \right)\cdot\left\langle\vec{u} \right\rangle,$ |
| --- | --- |

|  | $v_{\left\langle\vec{u} \right\rangle}\left( t \right)= \int_{\vec{u}} P\left( \vec{u} \vert\vec{I}_{u} \right)\vec{w}\left( t \right)\cdot\vec{u} d\vec{u},$ |
| --- | --- |

or

| (S11) | $v_{\left\langle\vec{u} \right\rangle}\left( t \right)=\int_{\vec{u}} P\left( \vec{u} \vert\vec{I}_{u} \right)v\left( t:\vec{u} \right) d\vec{u}.$ |
| --- | --- |

Therefore, $v_{\left\langle\vec{u} \right\rangle}\left( t \right)$ is the mean of $v\left( t:\vec{u} \right)$ over all $\vec{u}$. Consequently, if $v\left( t:\vec{u} \right)$ varies with $\vec{u}$, then sometimes $v\left( t:\vec{u} \right)>v_{\left\langle\vec{u} \right\rangle}\left( t \right)$ and sometimes ${v\left( t:\vec{u} \right)<v}_{\left\langle\vec{u} \right\rangle}\left( t \right)$, because otherwise, $v_{\left\langle\vec{u} \right\rangle}\left( t \right)$ would not be the mean of $v\left( t:\vec{u} \right)$. Hence, because sometimes $v\left( t:\vec{u} \right)>v_{\left\langle\vec{u} \right\rangle}\left( t \right)$, there are $\vec{u}$ whose associated value is larger than $v_{\left\langle\vec{u} \right\rangle}\left( t \right)$. Thus, when $v\left( t:\vec{u} \right)$ varies with $\vec{u}$, this inequality proves our claim. However, in two rare degenerate cases, $v\left( t:\vec{u} \right)$ does not vary with $\vec{u}$: First, in some rare cases, the $\vec{w}\left( t \right)$ at some moment $t$ and the structure of $P\left( \vec{u} | \vec{I}_{u} \right)$ could be such that $v\left( t:\vec{u} \right)$ would be constant as a function of $\vec{u}$. Second, in other rare cases, the $P\left( \vec{u} | \vec{I}_{u} \right)$ would be Dirac delta functions (Arfken and Weber, 1999). For these cases, $v\left( t:\vec{u} \right)\neq0$ for only one instance of $\vec{u}$. But even if such cases existed, they would be fleeting. Their conditions would disappear as $\vec{w}\left( t \right)$ continued to evolve.

*Comments on Claim 5*

- An implication of the existence of $\vec{u}$ such that $v\left( t:\vec{u} \right)>v_{\left\langle\vec{u} \right\rangle}\left( t \right)$ is that the most typical sensory signal is not the one eliciting most value. Thus, for example, an artist can produce pieces exaggerating aesthetic value.
- The degenerate case of the Dirac delta function is for a world in which only one sensory signal is possible. Such a world is not relevant for us.

*Claim 6*

Suppose that we replace the learning model in Eq. 1 by a nonlinear version

| (S12) | $v\left( m\left( t \right) ,\vec{w}\left( t \right),\vec{u}\left( t \right) \right)=m\left( t \right) V\left( \vec{w}\left( t \right),\vec{u}\left( t \right) \right).$ |
| --- | --- |

If $m\left( t \right)>0$ and $\nabla_{u}V\left( \vec{w}\left( t \right),\left\langle\vec{u} \right\rangle\right)\neq0$, then one can find an alternate sensory signal $\vec{u}_{a}\left( t \right)$ such that

| (S13) | $v\left( m\left( t \right),\vec{w}\left( t \right),\vec{u}_{a}\left( t \right) \right)> v\left( m\left( t \right),\vec{w}\left( t \right),\left\langle\vec{u} \right\rangle\right).$ |
| --- | --- |

*Proof*

The gradient of $v\left( m\left( t \right),\vec{w}\left( t \right),\vec{u}\left( t \right) \right)$ with respect to the components of $\vec{u}$ is

|  | $\nabla_{u}v\left( m\left( t \right) ,\vec{w}\left( t \right),\vec{u}\left( t \right) \right)=m\left( t \right) \nabla_{u}V\left( \vec{w}\left( t \right),\vec{u}\left( t \right) \right),$ |
| --- | --- |

and therefore,

| (S14) | $\nabla_{u}v\left( m\left( t \right) ,\vec{w}\left( t \right),\left\langle\vec{u} \right\rangle\right)\neq0.$ |
| --- | --- |

Because value increases along the direction of the non-zero gradient in Eq. S14, there exists a small enough $\varepsilon_{0}>0$ such that

| (S15) | $v\left( m\left( t \right) ,\vec{w}\left( t \right),\left\langle\vec{u} \right\rangle+{\varepsilon_{0}\nabla}_{u}v\left( m\left( t \right) ,\vec{w}\left( t \right),\left\langle\vec{u} \right\rangle\right) \right)>v\left( m\left( t \right) ,\vec{w}\left( t \right),\left\langle\vec{u} \right\rangle\right).$ |
| --- | --- |

Hence, if we define

|  | $\vec{u}_{a}\left( t \right)=\left\langle\vec{u} \right\rangle+{\varepsilon_{0}\nabla}_{u}v\left( m\left( t \right) ,\vec{w}\left( t \right),\left\langle\vec{u} \right\rangle\right),$ |
| --- | --- |

we prove Claim 6 from the inequality in S15.

*Comments on Claim 6*

- Violations of the condition $\nabla_{u}V\left( \vec{w}\left( t \right),\left\langle\vec{u} \right\rangle\right)\neq0$ will happen if $V$ is flat for sensory signals around $\left\langle\vec{u} \right\rangle$. Moreover, if $P\left( \vec{u} | \vec{I}_{u} \right)=0$ for the $\vec{u}$ around $\left\langle\vec{u} \right\rangle$, then we will also not be able to observe $\nabla_{u}V\left( \vec{w}\left( t \right),\left\langle\vec{u} \right\rangle\right)\neq0$. Most models avoid these conditions on $V$ and $P\left( \vec{u} | \vec{I}_{u} \right)$.
- The conditions $m\left( t \right)>0$ and $\nabla_{u}V\left( \vec{w}\left( t \right),\left\langle\vec{u} \right\rangle\right)\neq0$ are not rare. Therefore, broad classes of learning models allow for the possibility of exaggeration of value.
- The linear model in Eq. 1 is $V\left( \vec{w}\left( t \right),\vec{u}\left( t \right) \right)=\vec{w}\left( t \right)\cdot\vec{u}\left( t \right)$ and thus, $\nabla_{u}V\left( \vec{w}\left( t \right),\left\langle\vec{u} \right\rangle\right)=\vec{w}\left( t \right)$. Consequently, the linear model obeys the conditions of Claim 6 except when $\vec{w}\left( t \right)=0$. The proof of Claim 6 is thus an alternate proof of Claim 5.

References

Arfken, G. B. and H. J. Weber (1999). Mathematical methods for physicists, American Association of Physics Teachers.

Cheney, W. and D. Kincaid (2009). "Linear algebra: Theory and applications." The Australian Mathematical Society **110**.

Durrett, R. (2010). Probability: Theory and Examples. Cambridge University Press, Cambridge.

Horn, R. A. and C. R. Johnson (1990). "Norms for vectors and matrices." Matrix analysis: 313-386.

Karamardian, S. (2014). Fixed points, Elsevier.

Moya-Cessa, H. M. and F. Soto-Eguibar (2011). Differential equations: an operational approach, Rinton Press, Incorporated.

Strutz, T. (2010). "Data fitting and uncertainty." A practical introduction to weighted least squares and beyond. Vieweg+ Teubner.
